# Diverging Liquid–Liquid Phase Separation Behavior of Different Recombinant Major Ampullate Spidroins

**DOI:** 10.1002/advs.77295

**Published:** 2026-08-20

**Authors:** Tim Schiller, Cheryl Hayashi, Thomas R. Scheibel

**Affiliations:** ^1^ Chair of Biomaterials Engineering Faculty University of Bayreuth Bayreuth Germany; ^2^ Division of Invertebrate Zoology and Institute for Comparative Genomics American Museum of Natural History New York New York USA; ^3^ Bayreuth Center for Colloids and Interfaces (BZKG) Bayreuth Germany; ^4^ Bavarian Polymer Institute (BPI) Bayreuth Germany; ^5^ Bayreuth Center for Molecular Biosciences (BZMB) Bayreuth Germany; ^6^ Bayreuth Center for Material Science (BayMAT) Bayreuth Germany

**Keywords:** fiber, MaSp1, MaSp2, mechanical properties, spinning process

## Abstract

Spider silk fibers show exceptional mechanical properties, based on their underlying spider silk proteins (spidroins) as well as a highly developed spinning process, involving the transition of soluble high‐molecular‐weight spidroins into a hierarchically structured solid fiber. Liquid–liquid‐phase separation is crucial for the transition of the liquid to the solid state. Here, the assembly properties of a newly designed recombinant major ampullate spidroin 1 derivative based on an *Araneus* sequence have been investigated. Interestingly, the protein phase‐separates at slightly chaotropic conditions in contrast to respective MaSp2‐derived variants from *Araneus*. Fibers spun from the MaSp1‐derivative as well as MaSp1‐MaSp2‐mixtures provide a deeper insight into the assembly process of the spidroins and how hierarchical architectures of spider silk fibers could be achieved.

## Graphical abstract

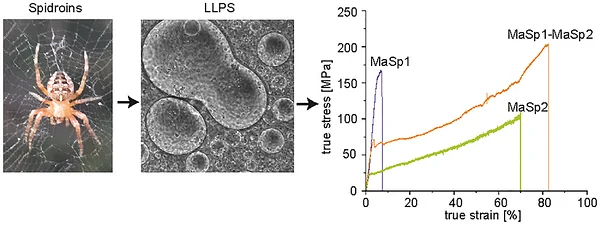

## 1 Introduction

Spider silk is one of the toughest natural fiber materials known. Among the various spider silk types, often up to seven in total, major ampullate silk is the most extensively studied [1]. Thus far, the detected silk proteins (= spidroins) of major ampullate silks are currently divided into five subtypes of Major ampullate spidroins (MaSps, MaSp1 to MaSp5), each thought to contribute in a different manner to the mechanical properties of the fiber [2]. MaSp1 forms crystalline β‐sheets in the mature fiber, which contribute to its high tensile strength. In contrast, the proline‐rich MaSp2 favors the formation of crystalline β‐spirals and β‐turns, providing extensibility to the silk fiber [3]. The presence of MaSp3, MaSp4, and MaSp5 has been correlated with increased fiber toughness [4]. MaSps are produced in distinctive glands that have three main parts: a tubular tail, a wide sac, and a narrow S‐shaped duct [5]. Studies of the gland tissue showed that the tail and sac contain three different types of epithelial cells in three different zones A, B, and C. The tail contains mainly zone A cells, which are also found in the part of the sac closest to the tail. The middle part of the sac contains zone B cells, while the part of the sac closest to the duct is composed of zone C cells [6]. It has been shown that spidroins are predominantly produced by zone A and zone B cells, whereas the proteins secreted by zone C cells still remain uncharacterized. This sequential protein production is further reflected in the hierarchical organization of the mature fiber [2, 7].

Within the silk gland, the proteins are stored in a liquid state as a highly concentrated dope which transforms along the spinning duct, influenced by several parameters, into a solid fiber [8, 9]. To describe the transition from soluble spidroins to a solid mature fiber, three different theories evolved over time. Vollrath and Knight established 1999 the liquid‐crystal‐theory, reporting that spidroins show characteristics typical for liquid crystals [10]. In 2003, Kaplan and Jin reported the formation of micelle‐like structures in regenerated *Bombyx mori* fibroin due to the block co‐polymer like structure of the fibroin, resulting in the micelle theory [11]. Coacervation or liquid–liquid phase separation (LLPS), as referred to here, is one other model to describe the transition from the liquid to the solid state [12, 13]. LLPS is a physical process of biomolecules (e.g. proteins) in solution, characterized by the spontaneous separation into two immiscible liquid phases, one with a low and one with a high concentration [14]. LLPS of spidroin solutions is triggered by external factors like changes in pH, temperature and salt concentration [13]. But also intrinsic features of the spidroins have a crucial impact on their LLPS behavior [15]. For instance, the overall architecture consisting of N‐ and C‐terminal domains (TDs) and a repetitive region is essential for LLPS. The TDs play a crucial role in fiber assembly [16]. The CTD of spider silk proteins has been shown to mediate pH‐dependent structural transitions essential for the formation of highly ordered silk fibers. This domain forms a parallel dimer and consists of a five‐helix bundle regulating the assembly and alignment of spider silk proteins. The CTD dimer is stabilized by salt bridges and in many spidroins additional by one or sometime two disulfide bonds and is primarily responsible for the solubility of the spidroins examined thus far [17]. Further, the CTD has been shown to be essential for proper LLPS of several spidroins [18, 19, 20].

The repetitive core region contains a sequence stretch folding into crystalline β‐sheet‐rich structures, predominantly formed by alanine residues. A more amorphous structure is formed by amino acids like glycine and serine in a second sequence stretch [21]. To describe the dependence of the LLPS behavior on amino acid composition, the amino acid motifs were classified as either stickers or spacers [20]. The amino acids belonging to stickers (e.g. tyrosine, arginine, glutamic acid and lysine), forming low‐affinity‐interactions like hydrogen bonding, π‐stacking or electrostatic interactions, and those which are spacers (e.g. alanine, glycine, proline) enable a dynamic, rapid adaption of the proteins, preventing aggregation [20].

Strikingly, although MaSp1 is a predominant component of dragline silk and has been used various times for artificial fiber spinning, its LLPS‐behavior has so far been sparely investigated [22]. Here, we designed a recombinant MaSp1‐derivative from a sequence of *Araneus gemmoides*, named eAGF1(O16) fused with the CTD NRC1 derived from *Latrodectus hesperus* and investigated its LLPS behavior. Fibers were wet‐spun from the highly concentrated protein dope followed by mechanical analysis, including a comparison to fibers from a previously established MaSp2‐derivative (eADF3((AQ)12)‐NRC1) [23, 24] as well as mixed MaSp1/MaSp2 fibers.

## 2 Materials and Methods

All chemicals and reagents were purchased from Carl Roth GmbH (Karlsruhe, Germany), if not otherwise stated. For preparation of buffer solutions, ultrapure water (MQ‐water‐systems, Merck KGaA; Darmstadt, Germany) was used.

### 2.1 Sequence Identification and Cloning of eAGF1(O16)‐NRC1

For sequence identification, the genome of *Araneus gemmoides* was analyzed for putative MaSp1 analogues, resulting in the identification of a repetitive region with seven slightly different amino acid sequence motifs. These sequence motifs were aligned and analyzed using Clustalω and Jalview. The resulting consensus sequence was named O‐module (GQGGAAAAAAAAGGQGGQGQYGLGAGQGYGAGLGGQGGS), which was repeated 16 times using our previously developed seamless cloning strategy [25]. In brief, the gene of interest was cloned into a pCS‐backbone for multimerization. The resulting eAGF1(O16) construct was extended with the sequence of the C‐terminal domain NRC1 derived from *Latrodectus hesperus* to yield the construct eAGF1(O16)‐NRC1. Finally, the gene was transferred to a pET29‐based vector for gene expression [25].

### 2.2 Production and Purification of the Recombinant Spidroins

The recombinant spidroins were produced via fed‐batch fermentation as reported previously [25]. For production, the pET29‐based expression‐vector was transformed into bacteria (*Escherichia coli* BL21‐gold). Protein production was induced adding 1 mM IPTG (Isopropyl‐β‐D‐thiogalactopyranoside) followed by fermentation in case of eAGF1(O16)‐NRC1 for 4 h at 30°C and 0.1 mM IPTG to produce eADF3((AQ)12)‐NRC1 overnight at 30°C. For purification, a previously published protocol was used [23, 25]. In brief, the cells were lysed using high‐pressure, afterwards a heat precipitation at 72°C was used to remove *E. coli* proteins, followed by protein precipitation using 10% ammonium sulphate. After lyophilization, the proteins were analysed by using SDS‐PAGE followed by silver staining, Western‐Blotting, CD‐spectroscopy and Fluorescence spectroscopy.

### 2.3 Circular Dichroism (CD)‐Spectroscopy and Fluorescence Spectroscopy

CD‐spectra (Jasco J‐715, Jasco, Germany) were recorded using a 0.1 cm path length quartz cuvette at a protein concentration of 0.1 mg/mL in 10 mM Tris/HCl, pH 8 at 20°C. The scan speed was 50 nm/min, step size 0.1 nm. Three scans were averaged. Fluorescence spectra (FP‐6200, Jasco, Germany) were recorded with a protein concentration of 0.5 mg/mL in 10 mM Tris/HCl, pH 8 at 20°C. For excitation of tryptophane, a wavelength of 295 nm, and for excitation of tyrosine, a wavelength of 280 nm was set.

### 2.4 SDS‐PAGE and Western‐Blot Analysis

SDS‐PAGE followed by silver staining and Western‐Blot analysis were performed as previously described [25]. For analysis, a HRP‐conjugated anti‐T7‐Tag antibody was used. For detection, the ECL plus‐kit (GE Healthcare) was used according to manufacturers’ recommendations.

### 2.5 Size Exclusion Chromatography Coupled to Multiangle Light Scattering (SEC‐MALS)

SEC‐MALS was conducted on a Superdex 200 Increase 10/300 GL‐column (Cytiva, Germany) connected to an Agilent 1100 Series HPLC system with a MALS detector DAWN EOS (Wyatt, Germany) to determine the molecular weights and protein sizes. The system was calibrated using BSA at a concentration of 5 mg/mL. Ultracentrifuged eAGF1(O16)‐NRC1 protein solution was applied at a concentration of ∼3 mg/ml via a 200 µL loop at a flow rate of 0.8 mL/min. In another approach, the protein solution was incubated with 10 mM DTT for 1 h at RT to reduce the disulfide bridge of the dimer. The eluted proteins were analyzed using a UV‐detector at 280 nm, a refractive index detector Shodex RI‐71, and a static light scattering detector DOWN EOS equipped with a K5 flow cell. Molecular masses and sizes were calculated from light scattering signals using the ASTRA 6 software (Wyatt, Germany).

### 2.6 Salting‐out Experiments and Atomic Force Microscopy (AFM) Analysis

To investigate the behavior of eAGF1(O16) (without NRC1) under kosmotropic conditions, the protein was dialyzed against 10 mM Tris/HCl, pH 8, and diluted to 1 mg/ml. Nanoparticle formation was triggered by adding 1 M potassium phosphate (KP_i_) buffer, pH 8 to a final concentration of 100 mM or 150 mM. Resulting nanoparticles were washed with MQ‐water and air‐dried.

Atomic force microscopy (AFM) analysis of dry nanoparticles was performed using a Nanosurf Flex Axiom with a C3000i Controller in Phase Mode and Si cantilevers (Tap190‐G, resonance frequency of 190 kHz, spring constant of 48 N/m; BudgetSensors Innovative Solutions Bulgaria Ltd.). Data were processed using Gwyddion software Version 2.68.

### 2.7 Liquid–Liquid‐Phase Separation (LLPS)

For LLPS, MaSp1‐derived eAGF1(O16)‐NRC1 and MaSp2‐derived eADF3((AQ)12)‐NRC1 were dissolved at a final concentration of 2.5 % (w/V) in 6 M guanidine thiocyanate (GdmSCN), each. To trigger phase separation, eAGF1(O16)‐NRC1 was dialyzed against 10 mM Tris/HCl, pH 8, and 200 mM NaCl at RT. After 1 h, the protein was centrifuged at 8500 rpm for 20 min at 4°C. After centrifugation, protein concentration of the two formed phases was measured upon diluting the high‐density phase with 8 M urea using a nanophotometer (NanoDrop; Thermo Fisher Scientific) at a wavelength of 280 nm and after zero compensation with the respective buffer. The molar extinction coefficient was calculated from the amino acid sequence using ExPASy ProtParam. The concentration of the high‐density phase ranged from 8% to 15% (w/V). To trigger phase separation in eADF3((AQ)12)‐NRC1, a previously established protocol was used [23]. In brief, the dissolved protein was dialysed against an aqueous buffer containing 50 mM Tris/HCl, pH 8.0, and 150 mM NaCl, followed by dialysis against 50 mM potassium phosphate buffer (pH 7.2) overnight at RT. The final solution was then centrifuged at 3,000 rpm and 4°C, and the supernatant was immediately separated from the high‐density phase. The concentration of the highly concentrated protein solution was measured using UV–vis spectroscopy (NanoDrop; Thermo Fisher Scientific). The final concentration of the dope ranged from 10% to 15% (w/V).

### 2.8 Particle Size Distribution Analysis

Particle size distributions were determined from light microscopy images (LeicaDMi8, Leica Microsystems CMS GmbH, Wetzlar, Germany) using ImageJ. Images were calibrated using the microscope scale bar, and individual condensates were manually measured using the line selection tool. For each sample, 200 condensates were analyzed from 10 independent images (20 condensates per image). The measured diameters were used to generate histograms and cumulative distribution functions (CDFs). The percentile diameters D_10_, D_50_, and D_90_ were determined from the CDFs and correspond to those particle diameters below which 10%, 50%, and 90% of the condensates were found, respectively. The span was used to quantify the breadth of the particle size distribution and was calculated using the following Equation (1):

(1) span=(D90−D10)/D50

### 2.9 Fiber Spinning

For fiber spinning, a wet‐spinning set‐up was used, consisting of a syringe pump (Nemesys, Cetoni GmbH Germany) and a Hamilton glass syringe (Hamilton Company, USA) connected to a polyethylene tubing (inner diameter: 0.38 mm; outer diameter: 1.09 mM, Smiths Medical, Germany). The dopes were extruded with flowrates ranging from 0.05 µL/s to 0.09 µL/s into a coagulation‐bath containing 80% isopropanol. The solid fibers were post‐stretched in 80% isopropanol to 150% and post‐treated in 100% ethanol. Afterwards, the fibers were air dried for further analysis.

### 2.10 Scanning Electron Microscopy (SEM)

For imaging the wet‐spun spidroin fibers, scanning electron microscopy was used (Volume Scope IKA Werke GmbH & Co KG). For sample preparation, the fibers were cut to pieces (0.5 mm to 1 cm in length) and fixed on a SEM‐stub using graphite tape. For better conductivity, aluminum tape was fixed on top of the samples, and the samples were sputter‐coated with 2 nm platinum (EM ACE600, Leica Microsystems).

### 2.11 Tensile Testing

All fibers were analyzed using an optical microscope (Leica DMI3000B, software Leica V4.3). Fiber diameters were monitored with a 20× object lens. From each fiber sample, several representative images were analyzed at different sites (*n* = 9) to measure fiber diameter and its standard deviation.

Upon analysis, fiber sections (n ≥ 5) were placed on plastic sample holders with a 2 mm gap using superglue (UHU GmbH Co. KG). Tensile testing was performed using a 50 N load cell (Tensile tester T300; MFC Sensortechnik) at a pulling rate of 0.005 mm s^−1^, 30% relative humidity and RT [26]. Mechanical properties were quantitatively evaluated using Excel (Microsoft) and Origin 9.4 (OriginLab Corporation, Northampton, MA, USA) considering true stress and true strain data. Stress‐strain curves were smoothed using the Savitzky‐Golay‐equation [26]. Statistical analysis was performed using Origin (OriginLab Corporation, Northampton, MA, USA). Differences between groups were assessed by one‐way ANOVA followed by Tukey's post hoc test. A p‐value < 0.05 was considered statistically significant. For the calculation of true strain (ε_t_) and true stress (σ_t_) the following equations were used:

(2) εt=ln(1+εe)

(3) σt=σe(1+εe)

ε_e_ = engineered strain; σ_e_ = engineered stress

### 2.12 Fourier‐Transformed Infrared (FTIR) Spectroscopy

FTIR spectra were recorded using a Bruker Tensor 27 (Ettlingen, Germany) with an attenuated total reflection (ATR) sampling technique on a germanium crystal. The spidroin after lyophilization and the fibers were characterized in solid state. For the characterisation of the dope, the highly concentrated spidroin solution was measured in wet state after blanking with the respective buffer. Spectra were recorded in absorbance mode at RT, with a 4 cm^−1^ resolution in the range of 900–4000 cm^−1^. OPUS software (version 6.5, Bruker Optik, GmbH) was used to process the data (corrected for background and atmosphere). Here, only the Amide I‐III range (1300–1800 cm^−1^) is shown, providing information concerning the protein secondary structure. Fourier self‐deconvolution (FSD) of the Amide I band was carried out as reported previously [27].

## 3 Results and Discussion

### 3.1 Design of Recombinant MaSp1 [eAGF1(O16)‐NRC1]

We designed a new recombinant spider silk variant termed eAGF1(O16), based on a consensus sequence derived from *Araneus gemmoides*. To the best of our knowledge, it is the first recombinant variant of an *Araneus*‐derived MaSp1 protein. The protein comprises 16 tandem repeats of the consensus sequence named O‐module with the amino acid sequence GQGGAAAAAAAAGGQGGQGQYGLGAGQGYGAGLGGQGGS. The consensus sequence was based on sequence alignment of seven identified sequence stretches within the natural genome of *Araneus gemmoides* (Figure 1a). The core sequence shows a hydrophilic stretch (GGQ‐motif) which is punctuated with hydrophobic amino acids (A = alanine, L = leucine) resulting in an alternating sequence in the Gly‐rich region. The repetitive core domain was extended with a previously identified CTD from *Latrodectus hesperus* named NRC1 [18], resulting in the recombinant spidroin eAGF1(O16)‐NRC1 with a molecular weight of ∼63 kDa. The recombinant eAGF1(O16)‐NRC1 was produced using *E. coli* and purified according to the established protocol by *Huemmerich* et al. [25]. In brief, *E. coli*‐derived proteins were thermally precipitated, followed by salting‐out of the recombinant spidroin using ammonium sulphate. The purity of the spidroin was analysed using SDS‐PAGE followed by silver staining and Western‐Blotting. Both analyses revealed a distinct protein band at ∼60 kDa representing eAGF1(O16)‐NRC1 (Figure 1B,C). Further, the molecular weight was analysed using SEC‐MALS, confirming the molecular weight of ∼63 kDa for monomeric eAGF1(O16)‐NRC1 and ∼126 kDa for the dimeric state (Figure S1). 1,4‐Dithiothreitol (DTT) was used to reduce the monomers, resulting in a slight increase in the hydrodynamic radius of the monomeric protein compared to the non‐reduced one. It was also observed that adding DTT did not result in complete monomerization of the dimers, aligning with the hypothesis that spidroin dimers are also structurally stabilized by non‐covalent interactions due to domain swapping [28] and as recently observed for naturally derived spidroins [29]. Since tryptophane residues are absent in the amino acid sequence of eAGF1(O16)‐NRC1, fluorescence emission upon excitation at 295 nm would indicate the presence of *E. coli* proteins. Fluorescence measurements of eAGF1(O16)‐NRC1 protein preparations revealed tyrosine emission spectra but no tryptophane fluorescence, confirming the purity of the silk protein (Figure 1D) [25].

**FIGURE 1:**
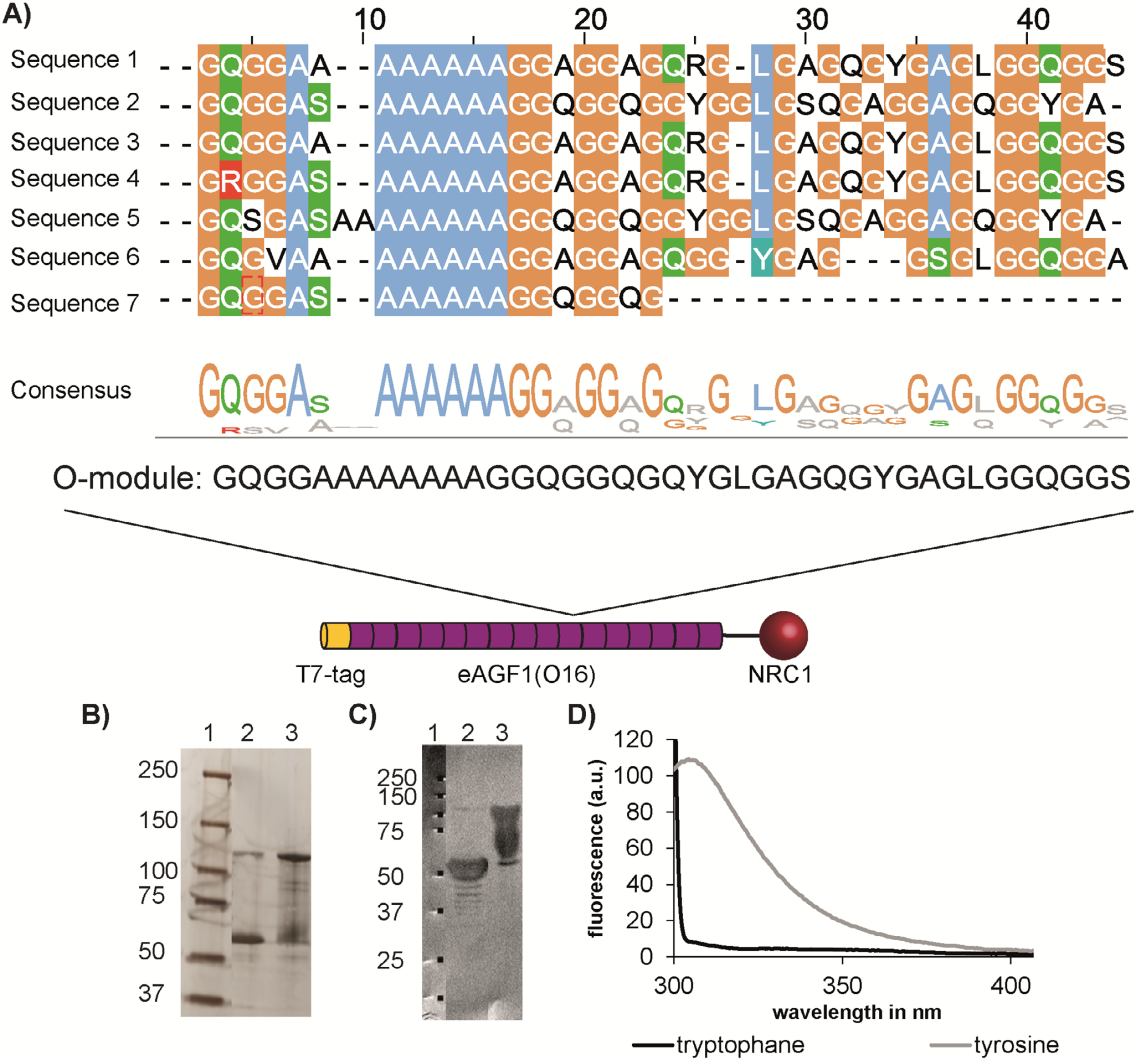
Design of an engineered recombinant MaSp1 variant. Amino acid sequence of the recombinant MaSp1 construct eAGF1(O16) used in this study. The sequence is derived from native MaSp1 of *Araneus gemmoides* and features 16 consensus sequence repeats. (A) The alignment of seven sequences derived from the genome of *Araneus gemmoides*, identified via BLAST searches, resulted in the design of the artificial amino acid sequence of the consensus sequence named O‐module. The recombinant eAGF1(O16) has been extended with the CTD NRC1 derived from *Latrodectus hesperus* resulting in eAGF1(O16)‐NRC1. (B) Silver staining of the purified recombinant spider silk protein eAGF1(O16)‐NRC1 showing a band at around ∼60 kDa under reducing conditions (lane 2) and a band at around 125 kDa under non‐reducing conditions (lane 3) representing the dimers of the protein. (C) Respective Western‐Blot of B) using a HRP‐conjugated anti‐T7‐antibody. (D) Fluorescence spectra of eAGF1(O16)‐NRC1 showing the emission spectra of tryptophane (black) and tyrosine (grey) residues, confirming the purity of eAGF1(O16)‐NRC1, since it does not contain any tryptophanes in contrast to putatively contaminating *E. coli* proteins.

The already established recombinant MaSp2 derivative eADF3((AQ12)‐NRC1 was analysed in comparison using SDS‐Page showing a protein band at ∼60 kDa under reduced conditions and at ∼120 kDa under oxidized conditions, representing the dimer (Figure S2A). Similar to eAGF1(O16)‐NRC1, fluorescence spectroscopy confirmed the absence of *E. coli* proteins (Figure S2B). However, for both spidroins, additional protein bands at lower molecular weights were observed, derived from shorter fragments of the recombinant protein, which are typically occurring in spidroin production upon pre‐final translation termination and partial degradation of the intrinsically unstructured core domains of the recombinant proteins. Similar observations have been reported previously for recombinant spidroins and are generally attributed to their large molecular size and highly repetitive sequence. In particular, the alanine‐ and glycine‐rich repeat regions pose challenges for recombinant expression, often resulting in premature translation termination and the production of truncated protein variants [30, 31, 32].

### 3.2 Liquid–Liquid‐Phase Separation Triggered by Slightly Chaotropic Ions

In general, the LLPS‐behavior of spidroins has been shown to play a crucial role during the fiber assembly process [13]. Within this process, the sticker‐spacer model is often used to describe residue‐residue interactions driving LLPS [20, 33]. According to this model, eAGF1(O16)‐NRC1 contains a hydrophobic polyalanine stretch (24.6%) being responsible for β‐sheet formation, important for final fiber formation and attributed to the spacers in terms of LLPS [20]. It also contains 40.0% of glycine residues being attributed to the spacers as well. The sticker regions are predominantly formed by tyrosine residues representing 4.8% of the sequence, Tyrosine residues usually range between 3.4% and 6.9% amongst spidroins [13]. Aromatic amino acids are thought to play a key role as stickers, triggering LLPS via π‐π stacking [20]. Recently, it was found that tyrosines are also involved in the formation of π‐cation interactions triggering LLPS‐behavior [34]. Also, glutamine residues are known to be relatively overrepresented in LLPS‐associated proteins. The recombinant eAGF1(O16)‐NRC1 contains 14% of glutamines, making it the third most abundant amino acid after glycine and alanine.

For the LLPS‐response not only the repetitive regions are essential, the CTD is also a prerequisite. The CTD regulates the conversion of liquid droplets into solid fibers [35]. The predicted LLPS behavior resulted in a probability of droplet‐promoting (P_DP_) value of 1.0 for both spidroins, eAGF1(O16)‐NRC1 and eADF3((AQ)12)‐NRC1. This residue‐level analysis suggested that both proteins possess a strong propensity to undergo liquid–liquid phase separation (LLPS) (Figure S3B) [36, 37, 38]. The CD spectra of both spidroins (Figure S3C) exhibited the structural features expected for spidroins comprising a CTD, with minima at approximately 205 and 222 nm, respectively [25]. The previously reported CTD NRC1 displays a characteristic α‐helical spectrum with minima at 208 and 222 nm [18], while the repetitive core domain eADF3((AQ)12) exhibits a minimum at ∼198 nm, characteristic of a random coil conformation [25]. Consequently, the CD spectra of the recombinant spidroins represent a superposition of these distinct structural contributions from the α‐helical CTD and the disordered repetitive core domain.

MaSp2‐like proteins derived from *Araneus diadematus* like eADF3 and eADF4 are known to be triggered by multivalent, kosmotropic ions such as phosphate, citrate or sulfate [19, 23]. For eADF3((AQ)12)‐NRC1 it is known that the presence of 50 mM potassium phosphate (KP_i_) triggers LLPS (Figure 2A) [23, 26]. Surprisingly, the MaSp1‐derived eAGF1(O16)‐NRC1 showed LLPS in presence of slightly chaotropic sodium chloride at concentrations ranging from 150 to 300 mM (Figure 2B). These conditions are similar to those found in the storage respiratory of the natural spider major ampullate gland [39, 40]. It has been widely reported that NaCl promotes liquid–liquid phase separation (LLPS) of intrinsically disordered proteins (IDPs) by modulating electrostatic interactions through ionic charge screening. This screening effect facilitates closer intermolecular contacts and enhances attractive interactions, such as hydrophobic, *π–π*, and cation*–π* interactions, which contribute to condensate formation. This formation results in the coexistence of a protein‐rich condensed phase (high density phase, HDP) and a protein‐poor dilute phase (low density phase, LDP). This effect is influenced by the salt concentration, the amino acid sequence, and the balance between attractive and repulsive intermolecular forces [41, 42, 43]. Since there are no basic or acidic residues in the here used spidroin sequences (Figure 2A,B), the effect of ionic charge screening was apparently neglectable.

**FIGURE 2:**
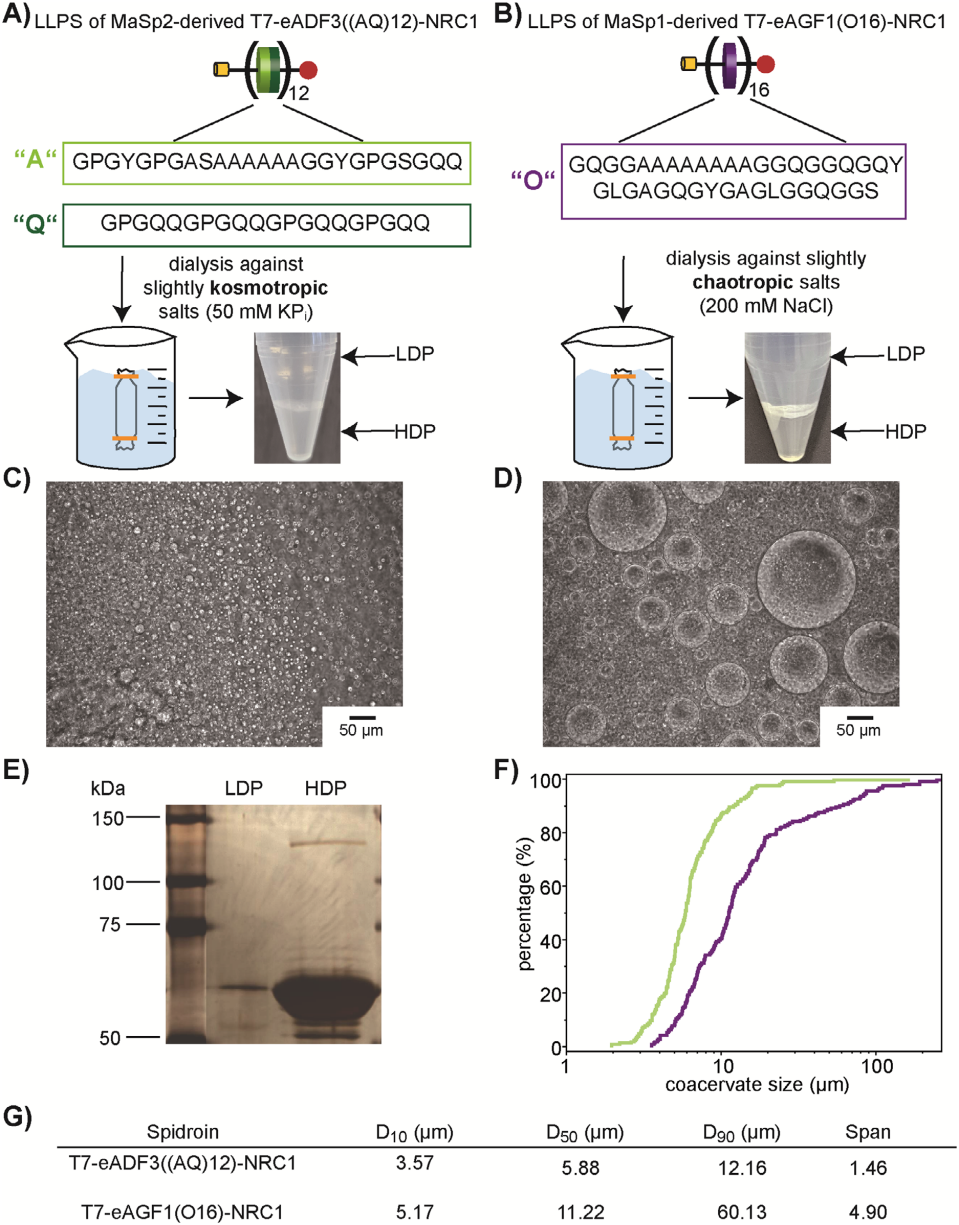
Analysis of the LLPS‐behavior of MaSp1‐derived eAGF1(O16)‐NRC1 in comparison to the previously established MaSp2‐derivative eADF3((AQ)12)‐NRC1. (A) Liquid–liquid phase separation behavior of the MaSp2 variant eADF3((AQ)12)‐NRC1 under slightly kosmotropic conditions (50 mM KPi). (B) Liquid–liquid phase separation behavior of eAGF1(O16)‐NRC1 under slightly chaotropic conditions (200 mM NaCl). (C) Light microscopy image of the condensates of eADF3((AQ)12)‐NRC1 formed during LLPS. (D) Light microscopy image of the condensates of eAGF1(O16)‐NRC1 formed during LLPS. (E) Reducing SDS‐PAGE of 0.8 µL aliquots of the low‐density phase (LDP; ∼5 mg/mL) and the high‐density phase (HDP, ∼150 mg/mL) of eAGF1(O16)‐NRC1. HDP was applied using a 1:10 dilution. (F) Cumulative distribution function (CDF) providing evidence for a narrower coacervate size distribution for eADF3((AQ)12)‐NRC1 in comparison to eAGF1(O16)‐NRC1. (G) The percentile diameters D_10_, D_50_ and D_90_ confirming larger diameters for eAGF1(O16)‐NRC1. Also, the calculated span of 4.90 indicates a broader distribution for eAGF1(O16)‐NRC1 than for eADF3((AQ)12)‐NRC1 (span: 1.46).

Light microscopy analysis revealed the formation of condensates for both engineered recombinant spidroins under the respective conditions (Figure 2C,D). Interestingly, the condensates formed by eAGF1(O16)‐NRC1 appeared larger in diameter than the ones formed by eADF3((AQ)12)‐NRC1. Further, fusion events of the droplets were observed for eAGF1(O16)‐NRC1 (supplementary movie S1). This property is typical for liquid‐like condensates and a property of LLPS [44]. SDS‐PAGE analysis of the two phases formed by eAGF1(O16)‐NRC1 revealed that the MaSp1‐derived protein partitioned into a HDP with very high protein concentrations and a LDP with a low protein concentration (Figure 2E). This behavior has been reported for MaSp2‐derived variants as well, but at quite different conditions as stated above [19]. To further analyse the distribution of the condensates, the diameters of the droplets were measured based on microscopy images. The distribution of the individual coacervate sizes is presented in the histograms, with eAGF1(O16)‐NRC1 showing a broader size distribution than eADF3((AQ)12‐NRC1 (Figure S4). For comparison, a CDF plot is shown in Figure 2F.

eADF3((AQ)12)‐NRC1 (green curve) formed smaller condensates than eAGF1(O16)‐NRC1 (purple curve), as evidenced by the leftward shift of the cumulative particle size distribution in the CDF. Furthermore, the shallower slope of the CDF for eAGF1(O16)‐NRC1 indicated a broader particle size distribution, whereas the steeper slope for eADF3((AQ)12)‐NRC1 reflected a narrower and more homogeneous distribution. Based on the CDF, the percentile diameters D_10_, D_50_, and D_90_ were determined (Figure 2G). These values correspond to the particle diameters below which 10%, 50%, and 90% of the particles are found, respectively. Overall, eAGF1(O16)‐NRC1 exhibited larger characteristic particle diameters than eADF3((AQ)12)‐NRC1. In addition, the calculated span was used to quantify the breadth of the particle size distribution. eADF3((AQ)12)‐NRC1 exhibited a span of 1.46, indicating a relatively narrow size distribution, whereas eAGF1(O16)‐NRC1 showed a span of 4.90, consistent with a substantially broader distribution of condensate sizes. In the prescence of potassium phosphate (KP_i_) concentrations ranging from 100 to 150 mM, eAGF1(O16) formed nanoparticles (Figure S5), in contrast to MaSp2‐derived spider silk proteins only forming nanoparticles at KP_i_ concentrations higher than 300 mM [45, 46].

### 3.3 Artificial Fiber Spinning and Mechanical Properties

The natural spidroin assembly process is highly complex involving several parameters for the transition of soluble spidroins to a solid fiber (Figure 3A). Epithelial cells covering the tail and parts of the ampulla (zone A) of the major ampullate silk gland produce the spidroins. Within the ampulla, the spidroins are stored in a soluble pre‐assembled state at high concentrations (up to 50 % (w/V)) in the presence of sodium and chloride ions (zone B) at neutral pH [40]. From the rear end of the ampulla (zone C), the spinning dope passes into an S‐shaped tapered duct which is lined by a cuticular intima layer [2]. This layer is hypothesized to function as a dialysis membrane, enabling the dehydration of the spinning dope [10]. Within the duct, sodium and chloride ions are replaced by the more kosmotropic potassium and phosphate ions inducing salting‐out of the proteins [8, 47]. Acidification (from pH 7.2 to 5.7) [48] leads to the formation of a quasi‐endless spidroin network due to conformational changes of the terminal domains [28, 49]. Finally, water resorption and shear stress, resulting from the pulling of the fiber from the spider's abdomen, lead to the final alignment of the spidroins followed by solidification of the fiber.

**FIGURE 3:**
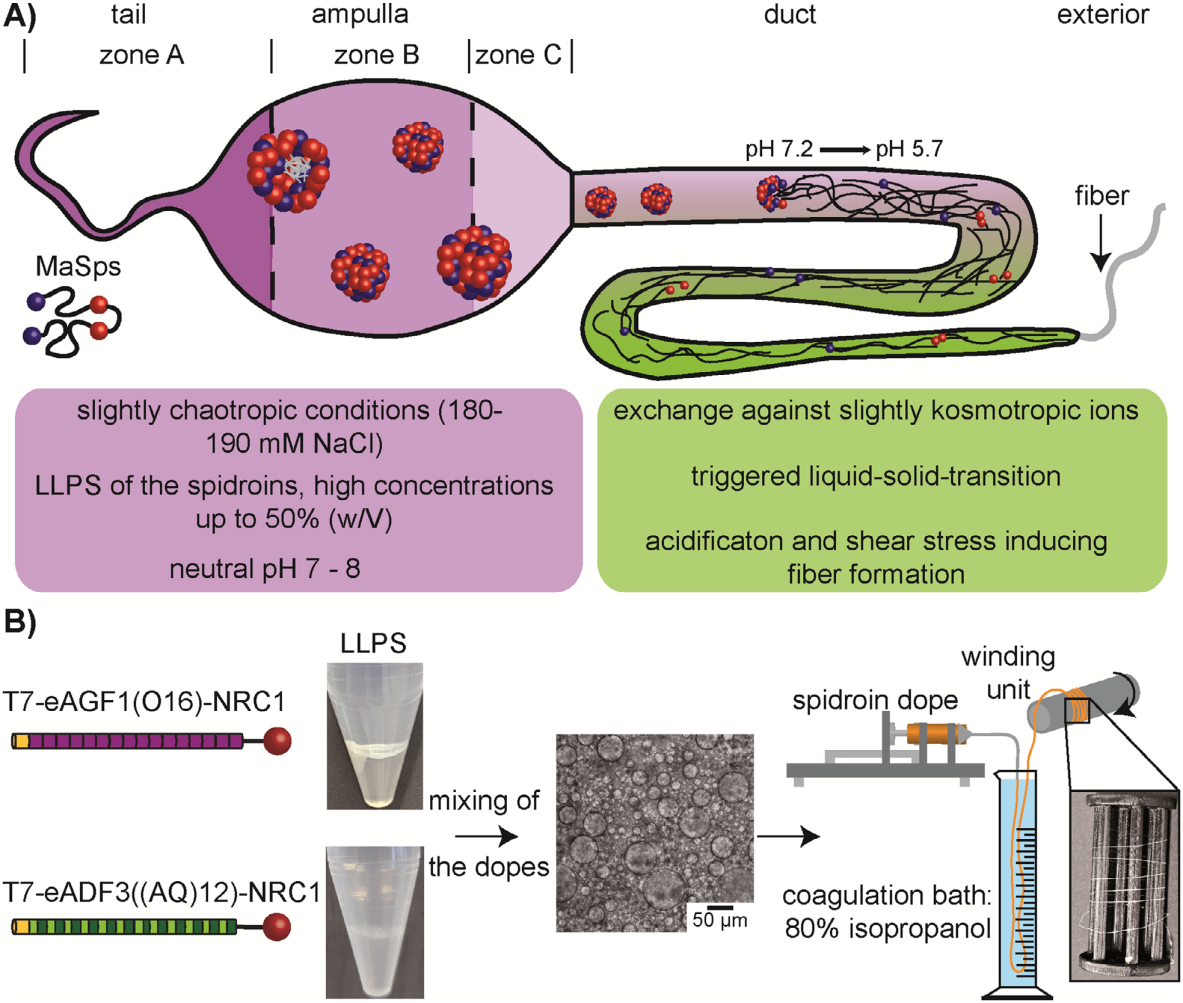
Natural spinning process in comparison to artificial wet‐spinning of a mixture of the recombinant spider silk proteins eAGF1(O16)‐NRC1 and eADF3((AQ)12)‐NRC1. (A) Schematic of a silk gland showing that in spiders spidroins are secreted in the tail of a silk gland and likely undergo liquid–liquid phase separation yielding micelle‐like droplets allowing the storage at high concentrations (∼50% (w/V)) within the ampulla under slightly chaotropic conditions (∼180 mM‐190 mM NaCl). In the duct, NaCl is exchanged with slightly kosmotropic KP_i_, and additionally a pH shift occurs. Upon elongational flow, a fiber is formed and pulled out of the spinneret. (B) Mixing of the derived HDP of the two phase‐separated recombinant spidroins. Light microscopy analysis of the new dope revealed a mixture of condensates showing the sizes previously identified for the individual proteins. The cartoon shows the artificial spinning set‐up consisting of a syringe filled with the dope, a syringe pump, a coagulation bath of 80% isopropanol and a winding unit.

Next, we wanted to identify the effect of the divergent LLPS behavior on artificially spun fibers. For artificial fiber spinning, the recombinant spidroins were first phase separated to yield highly concentrated dopes. While, as mentioned above, eAGF1(O16)‐NRC1 underwent LLPS under slightly chaotropic conditions (200 mM NaCl), mimicking the conditions within the ampulla (∼180 ‐190 mM NaCl) [39, 40], eADF3((AQ)12)‐NRC1 phase‐separated under slightly kosmotropic conditions (50 mM KPi) similar to the environment in the spinning duct (Figure 3A,B). FTIR analysis of the soluble MaSp1‐ and MaSp2‐derived spidroins after lyophilization showed a maximum of the amide I band at around 1650 cm^−1^ (Figure S6A). Fourier‐self‐deconvolution (FSD) revealed a β‐sheet content of ∼10% (Figures S6D and S7A). Upon LLPS, a difference in β‐sheet content was observed (Figure S6B). Whereas the MaSp1‐based spidroin maintained the predominant random coil structure, the MaSp2‐based variant showed an increase of the β‐sheet content to ∼20% (Figures S6D and S7B). These differences in β‐sheet content within dopes have been already previously reported for *Bombyx mori* fibroin in dependence on the ion composition within the fibroin's environment. *Eliaz* et al. dissected natural silk glands and analyzed different sections. Within the ampulla, the presence of chaotropic salts led to the formation of microcompartments, preserving the native, random‐coil secondary structure of the fibroin. In contrast, within the duct, kosmotropic salts promoted the formation of nanocompartments with a higher β‐sheet content [50].

After LLPS, the high‐density phases were used for artificial wet‐spinning to produce fibers. The wet‐spinning set‐up consisted of a syringe pump extruding the dope into a coagulation‐bath (80% 2‐propanol) and a winding unit (Figure 3B). Biomimetic spinning was not possible, since eAGF1(O16)‐NRC1 immediately started to aggregate upon addition of KP_i_. To evaluate the impact of the underlying spidroin on the mechanical properties of wet‐spun fibers, the MaSp1‐ and the MaSp2‐derived protein were spun individually into fibers, and also a 1:1 mixture of the two different dopes was used for fiber spinning (Figure 3B). After mixing both phase‐separated proteins, light microscopy revealed that both condensate types represented by different diameters were present within the dope. The different wet‐spun fibers were then morphologically analysed using SEM. The obtained fibers showed diameters in the µm‐range (Figure 4A–C), which is typical for wet‐spun spidroin fibers [51]. Further, it was observed that eAGF1(O16)‐NRC1‐based fibers (Figure 4D) showed a rougher surface in comparison to eADF3((AQ)12)‐based ones (Figure 4E). The fibers based on the protein mixture showed a rougher surface and a porous structure (Figure 4F). This morphology is likely specific to the present processing conditions. Natural spider silk fibers typically exhibit a hierarchical fibrillar structure rather than a dense morphology [52]. However, a highly porous fiber structure is not commonly reported for native spider silk fibers. The observed porosity could be attributed to inhomogeneous mixing of the two highly concentrated and highly viscous spinning dopes. This effect could be caused by entrapped air within the dopes and could be mitigated, for example, by vacuum degassing prior to spinning.

**FIGURE 4:**
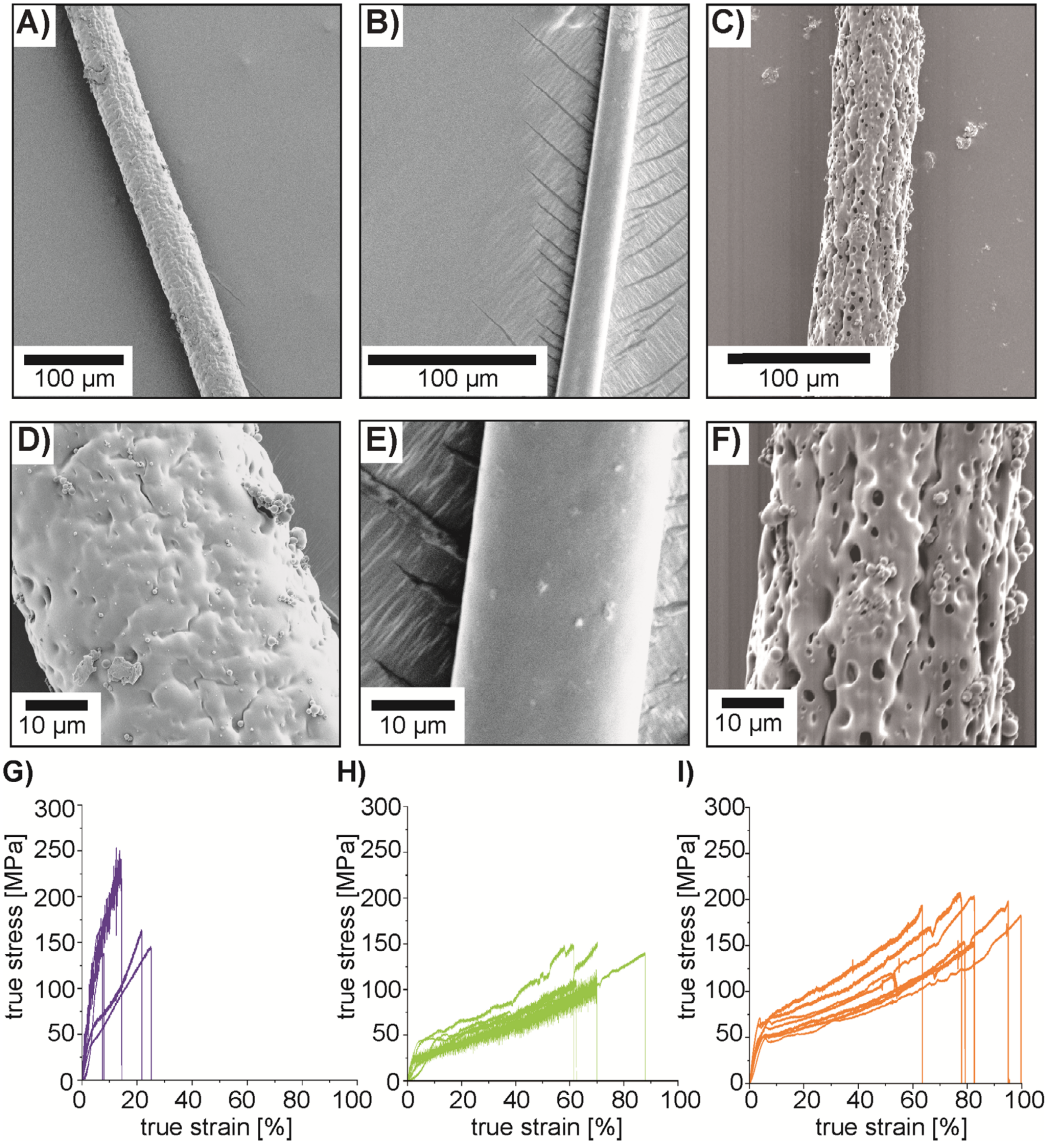
Morphological and mechanical analysis of the different spidroin‐based fibers. Scanning electron microscopy images of the spidroin fibers (A) eAGF1(O16)‐NRC1; (B) eADF3((AQ)12)‐NRC1; (C) mixture of eAGF1(O16)‐NRC1 and eADF3((AQ)12)‐NRC1. (D) Higher magnification of A). (E) Higher magnification of B). (F) Higher magnification of (C). True‐stress‐true‐strain curves of (G) eAGF1(O16)‐NRC1 fibers; (F) eADF3((AQ)12)‐NRC1 fibers; (I) fibers of the spidroin mixture.

Fibers of both spidroins as well as the respective mixture showed a shift of the amide I band from 1650 to 1630 cm^−1^ (Figure S6C) representing an increase of the β‐sheet content to ∼30% (Figures S6D and S7C,D) [53]. The formation of β‐sheet‐rich structures is known to be important for the mechanical properties of the silk fibers [54].

Tensile testing of the wet‐spun fibers revealed an E‐modulus of 2.6 GPa, an extensibility of 15%, a strength of 174 MPa and a toughness of 14.7 MJm^−3^ for eAGF1(O16)‐NRC1 fibers (Table 1). In comparison, biomimetically spun fibers of a MaSp1‐based protein derived from *E. australis* showed a Young´s modulus of ∼2 GPa, engineered strain <10%, engineered strength of ∼35 MPa, and a toughness <1 MJm^−3^ [55]. Other wet‐spun fibers based on MaSp1 of *Trichonephila clavipes* showed a Young´s modulus of ∼4 GPa, engineered strength of ∼62.3 MPa, engineered strain 3.5%, and a toughness ∼1.6 MJm^−3^ [56]. The wet‐spun fibers based on the MaSp2‐derivative eADF3((AQ)12‐NRC1 showed a quite different behavior with a higher extensibility of 71% and a lower tensile strength of 145 MPa (Figure 4H). The toughness of eADF3((AQ)12)‐NRC1 fibers was ∼50 MJm^−3^ (Table 1). In line with their high extensibility, their Young's modulus was also lower than that of eAGF1(O16)‐NRC1 ones. The fibers composed of the protein mixture of MaSp1 and MaSp2 showed an increased toughness of ∼84 MJm^−3^ and an increased tensile strength of 185 MPa (Table 1). Statistical significance of the measured mechanical properties was evaluated and is presented in Figure S8.

**TABLE 1:** Mechanical properties of wet‐spun fibers based on eAGF1(O16)‐NRC1, eADF3((AQ)12)‐NRC1 and the respective mixture.

Protein	eAGF1(O16)‐NRC1	eADF3((AQ)12)‐NRC1	MaSp1‐MaSp2 mixture
fiber diameter	22.6 ± 6.5 µm	30.2 ± 8.2 µm	29.7 ± 9.2 µm
extensibility	15 ± 7%	71 ± 9%	83 ± 11%
strength	174 ± 45 MPa	145 ± 14 MPa	185 ± 22 MPa
toughness	14.7 ± 7.7 MJ m^−3^	49.5 ± 12.4 MJ m^−3^	83.6 ± 12.6 MJ m^−3^
Young's modulus	2.6 ± 1.1 GPa	1.1 ± 0.1 GPa	1.7 ± 0.5 GPa
sample number	n = 5	n = 5	n = 7

While the enhanced mechanical properties of the mixed fibers may arise from synergistic interactions between eAGF1(O16)‐NRC1 and eADF3((AQ12)‐NRC1, it is likely that the resulting fibers consist of alternating segments of eAGF1(O16)‐NRC1 and eADF3((AQ)12)‐NRC1, which primarily governs the observed mechanical response. Although both spidroins possessed comparable molecular weights (∼60 kDa) and were spun from dopes of identical concentration (15% w/v), subtle differences in solution viscosity cannot be excluded and may have influenced the resulting mechanical properties [57]. Furthermore, variations in molecular orientation during spinning could contribute to the observed differences, as improved alignment is often associated with increased strength and stiffness [58]. The presence of a larger number of interacting molecules in the mixed system may additionally facilitate network formation and stress transfer within the fiber. Finally, a pre‐structuring of eAGF1(O16)‐NRC1 induced by its sensitivity to potassium phosphate (KP_i_) could not be excluded and may influence its assembly behavior during spinning (Figure S9).

## 4 Conclusion and Outlook

We engineered a novel recombinant major ampullate spidroin 1 (MaSp1) based on a consensus sequence of *Araneus gemmoides*. Notably, this engineered spidroin variant exhibits liquid–liquid phase separation (LLPS) behavior in response to slightly chaotropic conditions, a phenomenon previously described only for miniMiSp from *Trichonephila* antipodiana [59], but reported here for the first time for MaSps. These conditions are very similar to that of the environment within the ampulla of the spider silk glands, enabling the storage of spidroins at high concentrations. In contrast, all previously characterized MaSps undergo phase separation at slightly kosmotropic salt conditions, which are found in the spinning duct of the major ampullate silk gland. One key aspect influencing the LLPS behavior of a protein is its amino acid sequence, such as the number and distribution of tyrosine residues in case of eAGF1(O16)‐NRC1. Importantly, it cannot be excluded that posttranslational modifications such as phosphorylations in vivo might also play a significant role in LLPS [60]. The amino acid sequence as well as putative posttranslational modifications do not only affect the LLPS behavior, they also affect the assembly of the spidroins [61], the resulting fiber mechanics [3], as well as the supercontraction behavior of silk fibers [55]. As predicted, the MaSp1‐derived variant contributed to fiber strength, whereas the MaSp2‐derived protein contributed to the fiber strain when fibers were spun out of a mixture of both, and this could be shown for the first time for a completely artificial set‐up (Figure 5) [3, 62].

**FIGURE 5:**
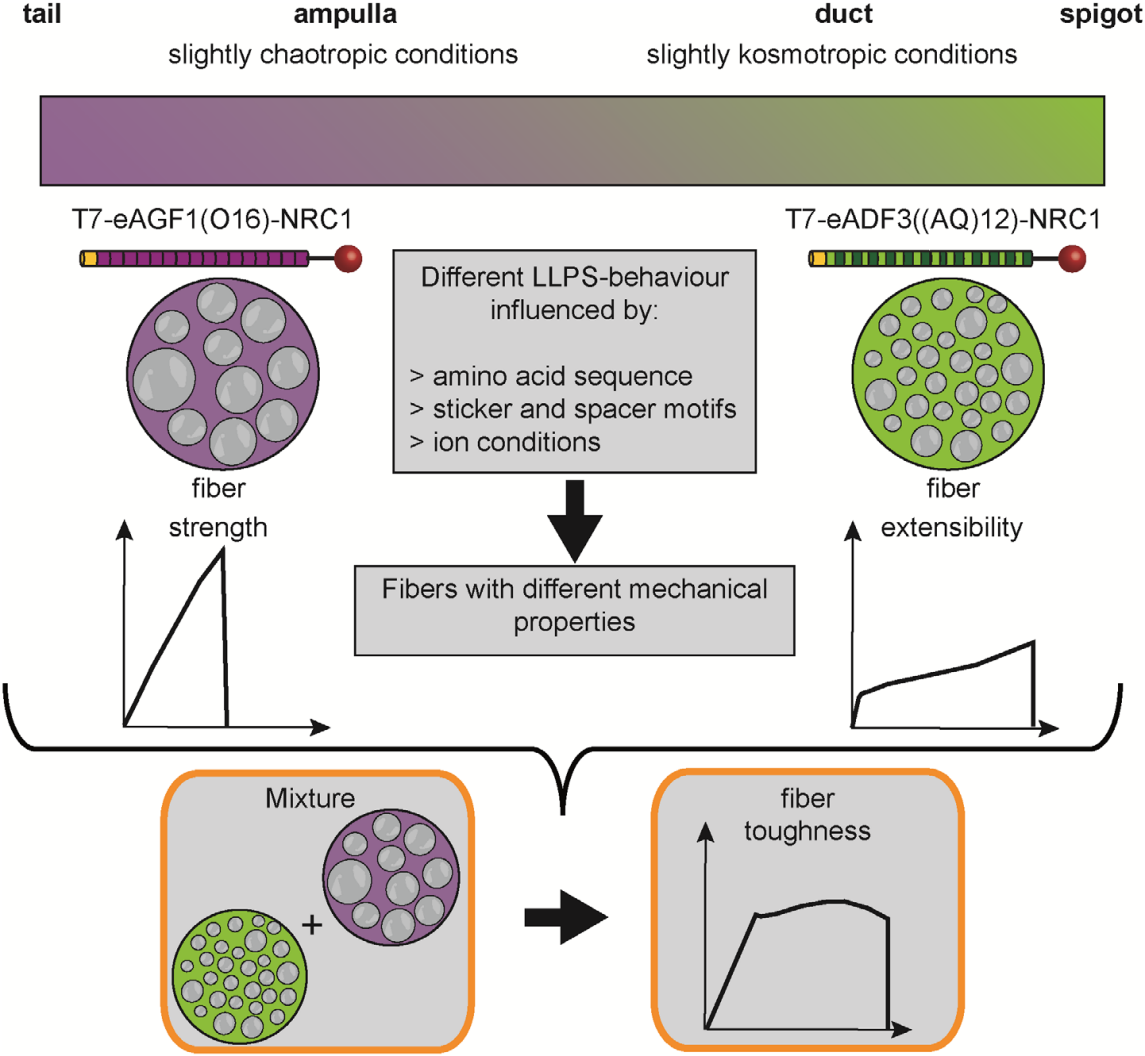
MaSp1‐ and MaSp2‐based proteins comprise different amino acid sequences, leading to different LLPS behavior due to the presence of different sticker and spacer motifs and in dependence of ion conditions in the environment. The identified LLPS behavior and the underlying amino acids are influencing the mechanical properties of the processed fibers, even upon non‐native fiber spinning conditions.

Our results reveal that contrary to the prevailing assumption that engineered MaSps typically undergo LLPS under slightly kosmotropic conditions, LLPS can also be induced by slightly chaotropic ions depending on the sequence of the respective MaSp. This finding adds one further level of control how to achieve hierarchical structures as found in spider silk fibers.

## Author Contributions

T.S. planned and conducted the experiments and further conceptualized and wrote the manuscript. C.Y.H. identified the natural protein sequences, performed sequence alignment, reviewed, and edited the manuscript. T.R.S. was responsible for funding acquisition, planning the experiments, conceptualization, finalizing the writing, and supervising the project. All authors have read and agreed to the published version of the manuscript.

## Conflicts of Interest

The authors declare the following conflicts of interest(s): T. R. S. is co‐founder and share‐holder of AMSilk GmbH. Tim Schiller and Cheryl Y. Hayashi declare that they have no competing financial interests or personal relationships that could have appeared to influence the work reported in the present paper.

## Supporting information




**Supporting File 1**: advs77295‐sup‐0001‐SuppMat.docx.

**Supporting File 2**: advs77295‐sup‐0002‐MovieS1.mp4.

## Data Availability

The data that support the findings of this study are available from the corresponding author upon reasonable request.
