# Supplementary material for: Diverging Liquid–Liquid Phase Separation Behavior of Different Recombinant Major Ampullate Spidroins
Source: Adv Sci (Weinh). 2026 Aug 20:e77295. Online ahead of print. doi: 10.1002/advs.77295 (PMC13491365; doi:10.1002/advs.77295)
Supplement: Supplementary file 1 [file ADVS-9999-e77295-s002.docx]

**SUPPORTING INFORMATION**

Diverging liquid-liquid phase separation behaviour of different recombinant major ampullate spidroins

Tim Schiller^1^, Cheryl Hayashi^2^, Thomas R. Scheibel^*1,3,4,5,6^

^1^Chair of Biomaterials, Engineering Faculty, University of Bayreuth

Prof.-Rüdiger-Bormann-Straße 1, 95447 Bayreuth, Germany

E-mail: [thomas.scheibel@uni-bayreuth.de](mailto:thomas.scheibel@uni-bayreuth.de)

^2^ Division of Invertebrate Zoology and Institute for Comparative Genomics,

American Museum of Natural History, New York, NY 10024, United States of America

^3^ Bayreuth Center for Colloids and Interfaces (BZKG)

^4^ Bavarian Polymer Institute (BPI)

^5^ Bayreuth Center for Molecular Biosciences (BZMB)

^6^ Bayreuth Center for Material Science (BayMAT)

**Figure S1: SEC-MALS analysis of eAGF1(O16)-NRC1 under reducing (purple) and non-reducing (pink) conditions.** SEC-MALS analysis confirmed the molecular weight (MW) of ~63 kDa for the monomeric eAGF1(O16)-NRC1 and ~126 kDa for the dimeric protein. The addition of DTT did not result in complete monomerization of the dimeric species. In addition, larger assemblies with a MW of ~160 kDa were observed.

**Figure S2: Analysis of eADF3((AQ)12)-NRC1**. A) Silver staining of the purified recombinant spidroin eADF3((AQ)12)-NRC1 sreveals a band at around ~60 kDa under reducing conditions (lane 2) and a band at around ~120 kDa under non-reducing conditions (lane 3) representing the dimer of the protein. B) Fluorescence spectra of eADF3((AQ)12)-NRC1 showing the emission spectra of tryptophane (black) and tyrosine (grey) residues, confirming the purity of eADF3((AQ)12)-NRC1, since it does not contain any tryptophanes in contrast to putatively contaminating E. coli proteins.

**Figure S3: Analysis of the recombinant spidroins eAGF1(O16)-NRC1 and eADF3((AQ)12)-NRC1.** A) Hydropathy-blots of both spidroins show the hydrophobic region (alanine-block) indicated by a positive score followed by a small hydrophilic part (negative score). The CTD adds a mostly hydrophobic section to the core sequences. B) Analysis of the LLPS propensity using FuzDrop.^1^ P_DP_-values ≥0.6 predict that a protein is likely able to undergo spontaneous liquid-liquid-phase-separation (LLPS). The overall P_DP_-value (based on a per-residue evaluation) for both spidroins (eAGF1(O16)-NRC1 and eADF3((AQ)12)-NRC1) is 1.0. C) Both CD-spectra of eAGF1(O16)-NRC1 and eADF3((AQ)12)-NRC1 show a minimum at ~205 nm and a second minimum at 222 nm indicating the mixture of the known α-helical structure of the NRC1-domain and the random coil structure of the repetitive core domain.

**Figure S4: Distributions of log-transformed coacervate diameters revealed by image analysis. Histograms represent the observed particle counts, and solid lines indicate the corresponding kernel density estimates.** The distribution of eAGF1(O16)-NRC1 (A) shows greater polydispersity and a larger fraction of larger condensates compared with the narrower distribution of eADF3((AQ12)-NRC1 (B). The condensate diameters are presented log-transformed due to the large distribution of eAGF1(O16)-NRC1 diameters.

**Figure S5: AFM imaging was used to analyze the behaviour of the repetitive core-domain eAGF1(O16) (in the absence of the CTD) at kosmotropic conditions (100 mM and 150 mM, respectively).** The addition of KP_i_ resulted in nanoparticle formation.

**Figure S6: FTIR Analysis of the secondary structure of the spidroins based on eAGF1(O16)-NRC1 (purple), eADF3((AQ)12)-NRC1 (green) and the respective mixture (orange).** A) FTIR spectra of the lyophilized spidroins showed a maximum peak of the amide I band at ~1650 cm^-1^. B) The dope made of eAGF1(O16)-NRC1 (purple) showed a peak of the amide I band at ~1650 cm^-1^. In comparison, the dope of eADF3((AQ)12)-NRC1 (green) showed a slight shift to lower wavenumbers. C) The fibers made of eAGF1(O16)-NRC1 (purple), eADF3((AQ)12)-NRC1 (green), and the respective mixture (orange) showed maxima at ~1630 cm^-1^, indicating a transition towards β-sheet structures. D) Fourier-self-deconvolution of the lyophilized proteins revealed a β-sheet content of ~10% for both spidroins. The eAGF1(O16)-NRC1-based dope showed also ~10% β-sheet content, whereas the eADF3((AQ)12)-NRC1-based dope showed an increase of the β-sheet content to ~20%. For all spidroin fibers an increase of the β-sheet-content to ~30% was observed.

**Figure S7: Exemplary band assignments chosen to calculate the β-sheet content of the different spidroin morphologies.** A) eAGF1(O16)-NRC1 and eADF3((AQ)12)-NRC1 in solution (after lyophilization). B) eAGF1(O16)-NRC1 and eADF3((AQ)12)-NRC1 in dope state. C) fibers spun individually of either eAGF1(O16)-NRC1 or eADF3((AQ)12)-NRC1 and D) fibers based on the spidroin mixture

**Figure S8: Mechanical properties of the eAGF1(O16)-NRC1-based (purple), the eADF3((AQ)12)-NRC1-based (green), and MaSp1-MaSp2-mixture-based (orange) fibers, obtained using tensile testing.** A) true stress, b) true strain, c) toughness, and d) Young’s modulus. The error bars represent the standard deviation. Stars indicate that the difference is significant (p-value ≤ 0.05, n ≥ 5).

**Figure S9: Extrusion of eAGF1(O16)-NRC1-based spinning solution in 50 mM KPi as coagulation bath.** A) Image of the formed aggregates within the coagulation bath. B) Zoom-in on the aggregates and fibrillar structures formed upon coagulation. Scale bars as indicated.

**Supplementary Movie S1**: Fusion event of eAGF1(O16)-NRC1-based droplets.

**References**

(1) Hardenberg, M.; Horvath, A.; Ambrus, V.; Fuxreiter, M.; Vendruscolo, M. Widespread occurrence of the droplet state of proteins in the human proteome. Proceedings of the National Academy of Sciences of the United States of America **2020**, 117 (52), 33254–33262. DOI: 10.1073/pnas.2007670117. Published Online: Dec. 14, 2020.
